# Computer-related ophthalmic syndrome in teachers of a University of the Province of Cañete

**DOI:** 10.1097/MS9.0000000000000177

**Published:** 2023-02-07

**Authors:** Yrene C. Uribe-Hernández, Fernando F. Ochoa-Paredes, Brian A. Meneses-Claudio, Carmen P. Tello-Aguilar, Roberto R. Buendía-Aparcana, Alex Pacheco

**Affiliations:** a Escuela Profesional de Contabilidad, Facultad de Ciencias Empresariales, Universidad Nacional de Cañete, Lima, Perú; b Escuela Profesional de Administración, Facultad de Ciencias Empresariales, Universidad Nacional de Cañete, Lima, Perú; c Universidad de Ciencias y Humanidades; d Escuela de Marketing y Dirección de Empresas, Universidad Cesar Vallejo, Lima, Perú; e Escuela Profesional de Ingenieria de Sistemas, Facultad de Ingeniería, Universidad Nacional de Cañete, Lima, Perú

**Keywords:** Cañete University, case report, diagnosis, education, eye diseases, patient population

## Abstract

Virtual education has impacted the vision of people during the coronavirus pandemic, as by spending more time on the computer, it compromises the eye health of the person causing long-term visual problems. So the objective of this investigation is to assess computer-related ophthalmic syndrome in teachers of a University of the Province of Cañete.

**Methods:**

This is a quantitative, nonexperimental, descriptive, cross-sectional study on a total population of 63 teachers, who answered a digital survey using the sociodemographic data and the Computer Vision Syndrome Questionnaire.

**Clinical Discussion:**

From the results it can be observed that the results of computer ophthalmic syndrome in the university teachers of the province of Cañete, where 51 (81%) of the teachers do not present the computer vision syndrome and 12 (19%) presented with the computer vision syndrome.

**Conclusion:**

The population conducting virtual education as well as the students should be educated on the measures to be taken to prevent computer ophthalmic syndrome and its consequences.

## Introduction

At the global level, after the outbreak of the coronavirus disease-2019 (COVID-19), the general population has been concerned about the long-term effects that the pandemic can generate, which can be considered high risk to one’s health and that they can affect mostly the young people, as they are not yet prepared to face this situation^1^.

It is not only health that is compromised, even the educational system is drastically compromised. Many of the researchers argue that resuming face-to-face teaching is uncertain, and that social distancing during the pandemic^2^ will have negative effects on the learning of young people, and that this will allow educational institutions to seek options to face this difficult situation and can continue educating the young^3^.

Likewise, many of the countries closed their educational institutions to avoid the spread of the virus in their younger population^4^, and due to this, they decided to carry out distance education through digital platforms to provide an education that is still of quality until the COVID-19 pandemic has subsided^5^.

At present, the educational system in the last decades has advanced considerably, and that have been shown to be immensely useful during the COVID-19 pandemic^6^, as with the support of digital platforms, it has been possible to maintain quality education^7^.

However, online education has had its consequences, as spending so much time on the computer has generated eye complications^8^, such as eye diseases, sleep disorders, inadequate posture, not only for students, but also for teachers, and the general population^9^,^10^.

For this reason, eye health was negatively affected during the COVID-19 pandemic owing to the online education. Several risks have been seen with prolonged computer use which, if not treated timely, can cause loss of vision^11^.

In a study conducted in India^12^ on 941 participants among university teachers, students, and the general population the prevalence of visual fatigue was higher in university students (50.6%) than in teachers and the general population (33.2%) and therefore university students tended to be at a high risk of computer vision syndrome.

In a study carried out in Saudi Arabia^13^ with the participation of university personnel, it has been observed that 81.2% of them tended to present computer vision syndrome and that 52.3% were women with a higher disease index. The main symptoms of the disease were dry eyes, headache, short vision, and difficulty concentrating.

In a study carried out in Egypt^14^ with the participation of university personnel who worked in the area of technological information, it was observed that 82.41% of the personnel in general tended to present the syndrome of computer vision and in terms of symptoms, 81.5% presented headache, 75.9% ocular burning, and 70.4% had blurred vision.

Therefore, the objective of the research is to determine computer-related ophthalmic syndrome in teachers of a university in the province of Cañete, which contributes to taking care of the well-being and health of people who spend many hours in front of the computer, which in turn is essential to the sustainable development of the province of Cañete.

## Methods

### Research type and design

The research is quantitative, in terms of its methodology it is descriptive, not experimental, and cross-sectional^15^.

### SCARE 2020

This case report has been reported in line with the Surgical CAse REport (SCARE) 2020 criteria in order to ensure the quality of the report, in addition to increasing the strength and transparency of case study reporting^16^.

### Research registry unique identifying number

The present work does not have a Research Registry unique identifying number as this is not the first time in humans.

### Population and sample

The total population is made up of 63 teachers who work in a University center in the province of Cañete

### Inclusion criteria

1. Teachers who have worked more than 2–3 years at the university.
2. Teachers who have voluntarily agreed to participate in the study.
3. Teachers who work using the computer for their virtual classes.

### Social history

1. Patients with no tobacco, alcohol, or recreational drug use.
2. Patients who are socially independent, have a driver’s license, and reside in a home of their own.

### Technique and instrument

A digital survey was carried out through the Google form, in which the Computer Vision Syndrome Questionnaire instrument was written. For data collection, it is carried out as follows: first, the sociodemographic aspects of age, if you are a user of glasses and time on the computer. Second, the Computer Vision Syndrome Questionnaire, which comprises 16 items distributed in two dimensions, of which the items are equal for both dimensions, and which determine the frequency and intensity of the symptoms at the ocular level; for the frequency of symptoms dimension, it is assessed with a Likert-type scale with three response options where: ‘1=Never,’ ‘2=Occasionally,’ and ‘3=Often.’ In the intensity of symptoms dimension, it is assessed with a Likert-type scale with two response options where ‘1=Moderate’ and ‘2=Severe’; the higher the score, the presence of computer vision syndrome in university teachers will be evidenced^17^.

The validity of the instrument to determine the computer vision syndrome was performed with the Kaiser–Meyer–Olkin sample adequacy measure, obtaining a coefficient of 0.802 (KMO>0.5), while the sphericity test of Bartlett obtained significant results (*χ* ^2^ approximately=580.816; gl=120; *P*=0.000). The reliability of the instrument was determined based on Cronbach’s alpha statistical test, the same one that obtained for all the items (*i*=16) a coefficient of 0.904 (*α*>0.8).

### Place and application of the instrument

A digital survey was carried out using computers and cell phones to determine if there is a presence of computer vision syndrome in university teachers in the province of Cañete.

First, he coordinated with the university professors so that they could be voluntarily present in the research study, and also that they were oriented about the study that is being carried out.

After guiding the teachers, the virtual surveys were carried out spending 15 min on each teacher; at the end of the surveys it was very satisfactory as the teachers were very accessible and were able to detail the results in the research.

### Follow-up

Patients were followed up 30 days after diagnosis at home via telephone consultation.

## Results

**Figure 1:**
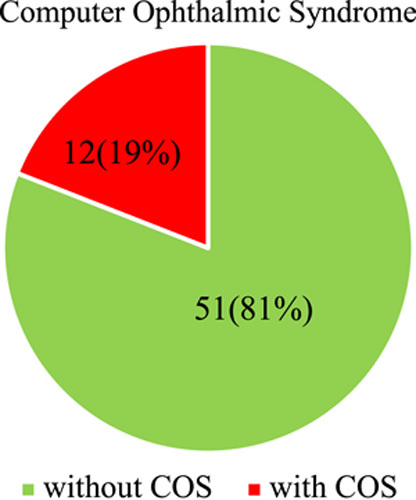
Computer ophthalmic syndrome (COS) in teachers of a University of the Province of Cañete.

Figure 1 shows the results of computerized ophthalmic syndrome in teachers of a University of the Province of Cañete, where 51 (81%) teachers do not have computer vision syndrome and 12 (19%) presented computer vision syndrome.

**Figure 2:**
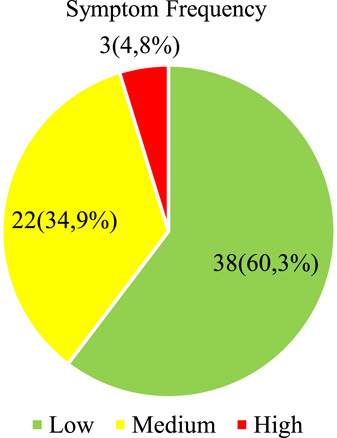
Computer ophthalmic syndrome: its dimension and frequency of symptoms in teachers of a University of the Province of Cañete.

Figure 2 shows the results regarding the frequency of symptoms in teachers, where 38 (60.3%) teachers present a low frequency of symptoms of computer ophthalmic syndrome, 22 (34, 9%) a medium frequency of symptoms, and 3 (4.8%) showed a high frequency of symptoms.

**Figure 3:**
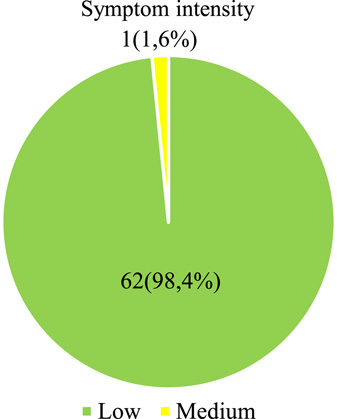
Computer ophthalmic syndrome: its dimension and intensity of symptoms in teachers of a University of the Province of Cañete.

In Figure 3, we observe that 62 (98.4%) teachers presented in low intensity the symptoms of computer ophthalmic syndrome and 1 (1.6%) in medium intensity the symptoms of computer ophthalmic syndrome.

Table 1 lists the computer ophthalmic syndrome and whether the teacher is a spectacle wearer, which was determined with Pearson’s *χ* ^2^ test. The significance level of the test obtained a value of 3.62 (*P*>0.05) (*χ* ^2^=0.187; DF=1). Therefore, we can interpret that 35 (79.5%) teachers who are users of glasses did not present the computer ophthalmic syndrome and 9 (20.5%) did present the computer ophthalmic syndrome; 16 (84.2%) teachers who are not users of glasses did not have computer ophthalmic syndrome and 3 (15.8%) of teachers who are not users of glasses had computer vision syndrome.

**Table 1:** Ophthalmic syndrome by computer in relation to whether the teacher uses lenses or not of a University of the Province of Cañete.

	Computer ophthalmic syndrome (COS)				
	Without COS	With COS	Total		
Are you a user of glasses?					
Yes					
ReCount	35	9	44		
% inside Are you a user of glasses?	79.5	20.5	100.0		
No					
ReCount	16	3	19		
% inside Are you a user of glasses?	84.2	15.8	100.0		
Total					
ReCount	51	12	63		
% inside Are you a user of glasses?	81.0	19.0	100.0		
*χ* ^2^ tests					
	Value	DF	Asymptotic significance (bilateral)	Exact significance (bilateral)	Exact significance (one-sided)
Pearson’s *χ* ^2^	0.187^a^	1	0.665		
Continuity correction^b^	0.007	1	0.934		
Likelihood ratio	0.193	1	0.661		
Fisher’s exact test				1.000	0.479
Linear by linear association	0.184	1	0.668		
No. of valid cases	63				

^a^ One cell (25.0%) has expected a count less than 5. The minimum expected count is 3.62.

^b^It has only been calculated for a 2×2 table.

Table 2 lists the computer ophthalmic syndrome and how long the teacher is at the computer, which was determined with Pearson’s *χ* ^2^. The significance level of the test obtained a value of 1.14 (*P*>0.05) (*χ* ^2^=1.486; DF=2). From which we can interpret that of the teachers who were at the computer from 2 to 4 h, 5 (83.3%) did not present ophthalmic syndrome and 1 (16.7%) did; from 4 to 8 h, 26 (86.7%) did not present computer ophthalmic syndrome and 4 (13.3%) did; of those who spent more than 6 h on the computer, 20 (74.1%) did not present ophthalmic syndrome and 7 (25.09%) did.

**Table 2:** Ophthalmic syndrome by computer in relation to how long is the teacher of a University of the Province of Cañete on the computer.

	Computer ophthalmic syndrome (COS)		
	Without COS	With COS	Total
How long is it on the computer?			
2–4 h			
ReCount	5	1	6
% within How long is it on the computer?	83.3	16.7	100.0
4–8 h			
ReCount	26	4	30
% within How long is it on the computer?	86.7	13.3	100.0
More than 6 h			
ReCount	20	7	27
% within How long is it on the computer?	74.1	25.9	100.0
Total			
ReCount	51	12	63
% within How long is it on the computer?	81.0	19.0	100.0
*χ* ^2^ tests			
	Value	DF	Asymptotic significance (bilateral)
Pearson’s *χ* ^2^	1.486^a^	2	0.476
Likelihood ratio	1.481	2	0.477
Linear by linear association	0.982	1	0.322
No. of valid cases	63		

^a^ Two cells (33.3%) have expected a count of less than 5. The minimum expected count is 1.14.

Table 3 lists the computer ophthalmic syndrome and whether the teacher has any visual disease, which was determined with Pearson’s *χ* ^2^. The significance level of the test obtained a value of 0.19 (*P*>0.05) (*χ* ^2^=2.487; DF=4). Therefore, it can be interpreted that 14 (82.4%) of the teachers with astigmatism do not present computerized ophthalmic syndrome, 3 (17.6%) of them do present it, and 8 (66.7%) of the teachers with myopia do not present computerized ophthalmic syndrome, 4 (33. 3%) do present it, 1 (100%) of the teachers with hyperopia do not present computerized ophthalmic syndrome, 2 (100%) of the teachers with cataract do not present computerized visual syndrome; and 26 (83.9%) of the teachers without disease do not present computerized ophthalmic syndrome and 5 (16.1%) do present it.

**Table 3:** Ophthalmic syndrome by computer in relation to if the teacher of a University of the Province of Cañete has any visual disease.

	Computer ophthalmic syndrome (COS)		
	Without COS	With COS	Total
Do you have any visual disease?			
Astigmatism			
ReCount	14	3	17
% within Do you have any visual disease?	82.4	17.6	100.0
Myopia			
ReCount	8	4	12
% within Do you have any visual disease?	66.7	33.3	100.0
Farsightedness			
ReCount	1	0	1
% within Do you have any visual disease?	100.0	0.0	100.0
Waterfall			
ReCount	2	0	2
% within Do you have any visual disease?	100.0	0.0	100.0
No sickness			
ReCount	26	5	31
% within Do you have any visual disease?	83.9	16.1	100.0
Total			
ReCount	51	12	63
% within Do you have any visual disease?	81.0	19.0	100.0
*χ* ^2^ tests			
	Value	DF	Asymptotic significance (bilateral)
Pearson’s *χ* ^2^	2.487^a^	4	0.647
Likelihood ratio	2.839	4	0.585
Linear by linear association	0.371	1	0.542
No. of valid cases	63		

^a^ Six cells (60.0%) have expected a count of less than 5. The minimum expected count is 0.19.

## Discussions

Computer ophthalmic syndrome is one of the conditions that develop due to prolonged use of computer, which is due to the educational measures promoted by various universities during the COVID-19 pandemic in order that their students do not get infected. But that, the concern today is as virtual classes are in a normalized way, it leads anyone to have a high exposure to electronic devices where they do their work or learn, which considerably affects one’s eye health, generating discomfort and visual alterations, damaging one’s well-being and health.

The prevalence of the computer ophthalmic syndrome that found in the study has been low, as well as in its dimensions. This is due to the fact that teachers are well at the level of their eye health; however, continuing with virtual classes may cause future problems in the quality of education, since not all teachers handle the technology, which is why virtual classes are increasingly carried out by young teachers who are able to handle it and who can also improve virtual education. With the users being younger the higher the prevalence to develop the syndrome in the long term. The study by Ganne *et al*.^12^ argues that eye problems are due to the fact that people’s predisposing time is so high that they generate the frequent symptoms of all ocular problems such as headache, dizziness, and constant blinking.

Regarding the teachers who spend more time at the computer, it is observed that the majority of teachers who spend from 2 to more than 6 h on the computer do not have ophthalmic syndrome, this is due to the fact that teachers who, above all, they are young they used measures they are young they used preventive measures such as massaging the eyes, fixing the gaze away from the screen and the use of artificial tears. It allowed a lower incidence of computer ophthalmic syndrome and that this allowed to continue carrying out virtual education for a longer time. In Mohamed *et al*.^14^, they argue that as the teacher performs more time in virtual education there is a risk that he will present with the ophthalmic syndrome, since by spending a longer time in front of electronic devices to work cuts the frequency of blinking, production of tear secretion, and leading to evaporation causing the symptoms of the syndrome.

Regarding the results of the presence of some visual disease and ophthalmic syndrome, we see that most do not present computer ophthalmic syndrome, although these visual alterations compromise ocular health in terms of focus and generate a visual effort of the person causing different ocular and visual discomfort. In Zalat *et al*.^13^ they argue that diseases such as astigmatism, myopia among others, are those that compromise the eye health of the person, generating the ophthalmic syndrome by computer, since the effort they make to be able to do their work, makes are more difficult and that allows you to see the risks that they can generate, such as constant eye fatigue, difficulty in seeing and loss of vision in one or both eyes.

## Conclusions

The population that carries out the virtual education as well as the students should be educated about the preventive measures taken to avoid computer-based ophthalmic syndrome and its consequences.

Strategies should be implemented that allow the prevention of ophthalmic syndrome in computer users and thus be able to maintain stable eye health.

The younger population should be made aware of why electronic devices should be used for less time and their risks to eye health.

## Ethical approval

Does not involve any type of ethical approval.

## Patient consent

Written informed consent was obtained from the patient for publication of this case report and accompanying images. A copy of the written consent is available for review by the Editor-in-Chief of this journal upon request.

## Sources of funding

This research work does not have any funding sources.

## Author contribution

The authors declare that they have participated in a coordinated and equal manner in each of the tasks involved in: (a) the original conception of the work; (b) review of the scientific literature; (c) analysis, acquisition, and interpretation of data; (d) drafting and critical revision of the content; and (e) in the final approval of the version to be published. Y.C.U.-H.: study concept; F.F.O.-P.: data collection; B.A.M.-C.: data analysis; C.P.T.-A.: interpretation; R.R.B.-A.: writing the paper; A.P.: writing the paper.

## Conflicts of interest disclosure

The authors declare no conflicts of interest.

## Research registration unique identifying number (UIN)

The present work does not have a Research Registry unique identifying number (UIN) as this is not the first time in man.

## Guarantor

A. Pacheco.

## Provenance and peer review

Not commissioned, externally peer reviewed.
